# Discrepancies in decision making preferences between parents and surgeons in pediatric surgery

**DOI:** 10.1186/s12911-021-01414-z

**Published:** 2021-02-04

**Authors:** Erica M. Carlisle, Caleb J. Klipowicz, Laura A. Shinkunas, Aaron M. Scherer, Lauris C. Kaldjian

**Affiliations:** 1 University of Iowa Carver College of Medicine, Iowa City, IA 52242 USA; 2 Program in Bioethics and Humanities, University of Iowa Carver College of Medicine, 200 Hawkins Drive 2966-Z-JPP, Iowa City, IA 52242 USA; 3 Department of Surgery, Division of Pediatric Surgery, University of Iowa Hospitals and Clinics, Iowa City, IA 52242 USA; 4 Department of Anthropology, University of Iowa, Iowa City, IA 52242 USA

**Keywords:** Decision-making, Urgency, Pediatric surgery

## Abstract

**Background:**

Little data exists regarding decision-making preferences for parents and surgeons in pediatric surgery. Here we investigate whether parents and surgeons have similar decision-making preferences as well as which factors influence those preferences. Specifically, we compare parents’ and surgeons’ assessments of the urgency and complexity of pediatric surgical scenarios and the impact of their assessments on decision-making preferences.

**Methods:**

A survey was emailed to parents of patients evaluated in a university-based pediatric surgery clinic and surgeons belonging to the American Pediatric Surgical Association. The survey asked respondents to rate 6 clinical vignettes for urgency, complexity, and desired level of surgeon guidance using the Controlled Preferences Scale (CPS).

**Results:**

Regarding urgency, parents were more likely than surgeons to rate scenarios as emergent when cancer was involved (parents: 68.8% cancer vs. 29.5% non-cancer, *p* < .001; surgeons: 19.2% cancer vs. 25.4% non-cancer, *p* = .051). Parents and surgeons were more likely to rate a scenario as emergent when a baby was involved (parents: 45.2% baby vs. 36.2% child, *p* = .001; surgeons: 28.0% baby vs. 14.0% child, *p* < .001). Regarding decision-making preferences, parents and surgeons had similar CPS scores (2.56 vs. 2.72, respectively). Multivariable analysis showed parents preferred more surgeon guidance when scenarios involved a baby (OR 1.22; 95% CI 1.08–1.37; *p* < 0.01) or a cancer diagnosis (OR 1.29; 95% CI 1.11–1.49; *p* < 0.01), and that both parents and surgeons preferred more surgeon guidance when a scenario was considered emergent (parents: OR 1.81; 95% CI 1.37–2.38, *p* < 0.001; surgeons: OR 2.48 95% CI 1.76–3.49, *p* < 0.001).

**Conclusions:**

When a pediatric patient is a baby or has cancer, parents are more likely then surgeons to perceive the clinical situation to be emergent, and both parents and surgeons prefer more surgeon guidance in decision-making when a clinical scenario is considered emergent. More research is needed to understand how parents’ decision-making preferences depend on clinical context.

## Background

Shared decision-making (SDM) has been highlighted as the preferred approach to clinical counseling due to its presumed promotion of patient autonomy, positive impact on quality of care, and reduction of healthcare costs [1]. SDM requires that physicians and patients work together to develop mutually agreed upon care plans that incorporate evidence-based standards of care and patient values and goals [2, 3]. Despite the perceived benefits of SDM, an emerging body of literature suggests that some patients may not always prefer extensive involvement in decision-making and seem to want more guidance from their physicians [4–7]. Moreover, it can be argued that the increased emphasis on SDM and patient autonomy in clinical counseling may prevent physicians from providing patients the guidance they desire when making decisions [6, 8].

Given this background, it is notable that physician attitudes toward SDM are relatively poorly understood. Available data generally suggests that physicians tend to support SDM in certain situations: when strong evidence for a given procedure is lacking; when evidence-based clinical guidelines do not support a particular treatment option; in non-emergent settings where patients are able to fully participate; and when treatment options are likely to significantly impact the patient’s life [9]. However, despite this degree of stated support, physicians often choose not to incorporate SDM into practice [9, 10]. The inconsistent support of SDM, by both patients and physicians, suggests that a physician-guided approach to decision-making may be a preferable approach to clinical counseling in some contexts.

The efficacy of SDM in pediatrics is not well defined [11–13]. This, in part, stems from challenges with engaging both parents and children (depending on age and maturity level) in the decision-making process[12, 13]. Difficulty also lies in the direct application of SDM models, which have typically been created for adult patients, to pediatric care where the decision maker is not a competent, adult patient but rather a surrogate decision maker (i.e. parent or legal guardian) who is tasked with making decisions in the best interest of the child [11, 14]. This contrasts with adult patients who are encouraged to make decisions consistent with their own values and priorities. It also contrasts with surrogate decision makers for adult patients who do not have decision-making capacity in that such surrogates are tasked with applying substituted judgement (i.e. what the patient would prefer if he or she were able to decide) as opposed to best interest standards [14]. These differences create challenges with direct application of SDM to pediatrics and have limited investigation of the efficacy of SDM in pediatrics [14, 15].

The value of SDM also remains relatively under-explored in surgery. This observation is especially significant when one considers how concerns about the appropriateness of SDM may be particularly pronounced in surgical settings that can entail irreversible outcomes that may be associated with a high risk of mortality or morbidity [7]. Consideration of such operations for pediatric patients may further heighten concern. Our recent work demonstrates that relatively little has been published on patient or surgeon preferences toward SDM in surgery [16, 17]. Those studies that do exist overwhelmingly focus upon elective, non-urgent decision-making [16, 17]. It is possible that patient and surgeon preferences toward the degree of surgeon guidance vary depending on the perceived urgency or complexity of a given setting. In our review of the literature, we did not find any studies that assessed this possibility [16, 17]. Most studies assume that patients and surgeons interpret the urgency and complexity of clinical situations similarly, however this has never been shown empirically. Discordant interpretation of the clinical setting may impact preferences toward the desired degree of surgeon guidance. Challenges with surrogate decision-making—as well as the complex decision-making triad that exists between patients, parents, and surgeons—may render SDM even more challenging in pediatric surgery than it is in adult surgery.

To investigate factors associated with decision-making preferences, we studied the attitudes of pediatric surgeons and parents of pediatric patients. We focused our investigation on whether parents and surgeons express similar decision-making preferences in pediatric surgery as well as which factors influence those preferences. Specifically, we first assessed whether there was congruity between parents’ and surgeons’ assessments of the level of urgency and complexity of specific clinical scenarios. We then explored the impact of parents’ and surgeons’ assessments about urgency and complexity on their respective attitudes toward decision-making and the degree of surgeon guidance they preferred.

## Methods

### Participants

In February/March 2019, all parents of patients who were seen in the University of Iowa Pediatric Surgery Clinic between 1.1.2014 and 12.31.2018 were sent an email invitation describing the study and containing a link to a web-based survey (Additional file 1: Appendix A). In April/May 2019, all members of the American Pediatric Surgical Association (APSA) who practice in the US were sent an email invitation describing the study and containing a link to a web-based survey that was analogous to the parent survey (Additional file 1: Appendix B). The present survey did not access APSA membership through established channels monitored by the APSA Outcomes and Evidence-Based Practice Committee, which may account for the lower response rate and may introduce bias into the results. Both parents and surgeons who did not respond to the initial email invitation were sent a follow-up invitation email 1 week later. Informed consent was obtained from all subjects, and all invited subjects were 18 years of age or older. Both surveys employed the Qualtrics survey platform (Proto, UT). The study was approved by the University of Iowa Institutional Review Board (IRB ID# 201808799), and all methods were conducted with the relevant guidelines and regulations. No incentives were provided for survey completion.

### Survey scenarios

Each survey consisted of six scenarios, presented in a randomized order, in which surgery was recommended for a pediatric patient. For all scenarios, surgical intervention is the generally accepted standard of care. Scenarios varied by diagnosis, urgency, complexity of procedure, and patient age. Scenarios included the following clinical issues: preterm baby with pneumoperitoneum; baby with recurrent ear infections; baby with cancerous lung mass; baby with benign lung mass; child with kidney cancer; and child with an asymptomatic inguinal hernia. To limit potentially confounding clinical variables, scenarios were purposely constructed to minimize extraneous detail and allow direct comparisons (i.e. baby with cancer as compared to child with cancer, baby with a cancerous lung mass as compared to baby with a benign lung mass, baby with an elective surgical need as compared to child with an elective surgical need). The scenario response options were pilot tested and critically reviewed by 10 clinicians (pediatric surgeons, pediatric surgery nurse practitioners, and pediatric surgery specialty nurses) for face validity and clarity.

### Measures

Respondents were asked whether they considered each scenario to be an emergency and whether they considered the recommended operation to be complex/difficult to perform. They also completed a modified Controlled Preferences Scale (CPS) [18] item to assess their decision-making preferences for each scenario. The CPS is a well-validated instrument that assesses desired level of physician involvement in decision-making (1 = autonomous patient decision-making, 2 = patient-led with surgeon input, 3 = shared decision-making, 4 = surgeon-led with patient input, 5 = decision made by surgeon). Surgeons were asked to complete a CPS item modified to assess their desired level of parent involvement in decision-making. All respondents were also asked to rank a list of factors considered important in determining if a surgery is an emergency. Demographic variables were collected.

### Data analysis

Survey responses were uploaded into SPSS 24.0 (Armonk, NY) for descriptive statistics, and we used Stata 14.2 (College Station, TX) for multivariable analysis. Two-tailed Fisher’s exact test or chi square tests were used to test for differences in proportions across scenarios. Aggregate CPS scores were created by averaging responses based on scenario characteristics (e.g. cancer/non-cancer) and compared using independent and paired samples t-tests, respectively.

Multivariable analysis was performed using mixed-effects logistic regression analysis to assess the impact of independent variables on urgency and complexity judgments and CPS scores while controlling for variability resulting from multiple responses from the same participant. Participant (parent or surgeon) was treated as a random effect in the analyses. Demographic characteristics were included in all analyses.

## Results

### Demographics

The parent survey response rate was 10.7% (239/2234). Parents had a median age of 38 years (range 19–62) and were predominantly white (95%), female (92%), privately insured (83%), and likely to have a Bachelor’s degree or higher (62%) (Table 1). The surgeon survey response rate was 10.8% (131/1211). Surgeons had a median age of 50 years (range 34–86) and were predominantly white (86%), male (73%), practiced in an academic institution (65%), and had practiced for > 10 years (55%). Surgeons reported near-equal geographic distribution across the continental US (Table 1).

**Table 1:** Respondent demographic information

Survey participant demographic information		
	Parents	Surgeons
Age	Median = 38 (19–62)	Median = 50 (34–86)
Race/ethnicity		
White	229 (95.8%)	112 (85.5%)
Non-white (any)	11 (4.6%)	23 (17.5%)
Gender identity		
Female	220 (92.1%)	32 (24.4%)
Male	15 (6.3%)	96 (73.3%)
Another gender identity not listed here	1 (0.4%)	1 (0.8%)
Highest degree of education		
Less than high school degree	1 (0.4%)	–
High school graduate (high school diploma or equivalent including GED)	18 (7.5%)	–
Some college but no degree	37 (15.5%)	–
Associate degree in college (2-year)	33 (13.8%)	–
Bachelor's degree in college (4-year)	86 (36.0%)	–
Master's degree	46 (19.2%)	–
Doctoral degree	8 (3.3%)	–
Professional degree (JD, MD)	9 (3.8%)	–
Insurance status		
Privately insured	198 (82.8%)	–
Medicare or Medicaid	36 (15.1%)	–
Uninsured	2 (0.8%)	–
Years of practice		
Less than 5 years	–	28 (21.4%)
5–10 years	–	29 (22.1%)
10–20 years	–	24 (18.3%)
More than 20 years	–	48 (36.6%)
Pediatric surgery fellow	–	1 (0.8%)
Inst. setting		
Academic	–	85 (64.9%)
Private practice	–	9 (6.9%)
Both academic and private practice	–	28 (21.4%)
Other	–	2 (1.5%)
Region		
Northeast	–	30 (22.9%)
Midwest	–	31 (23.7%)
West	–	28 (21.4%)
South	–	38 (29.0%)
Other (Alaska, Hawaii, Puerto Rico, US territories and affiliated Pacific Islands, etc.)	–	2 (1.5%)
Total	239 (100.0%)	131 (100.0%)

### Comparing parents’ and surgeons’ assessments of complexity and level of urgency

**Fig. 1:**
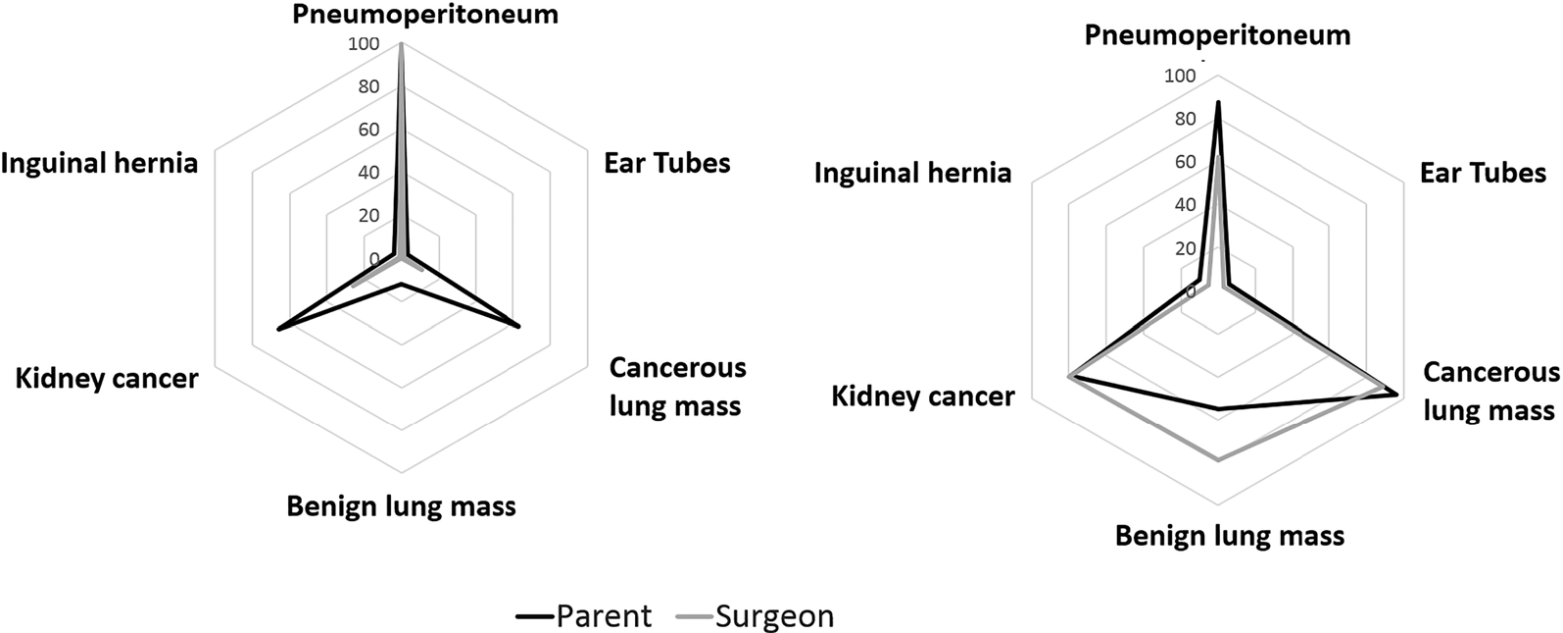
**a** Percentage of respondents who perceived each scenario to be emergent. **b** Percentage of respondents who perceived each scenario to be complex

As shown in Fig. 1a, b, most parents rated three scenarios as emergent (preterm baby with pneumoperitoneum, baby with cancerous lung mass, child with kidney cancer) and four scenarios as complex (preterm baby with pneumoperitoneum, baby with cancerous lung mass, baby with benign lung mass, child with kidney cancer). Most surgeons rated only one scenario as emergent (preterm baby with pneumoperitoneum), but like parents, they rated four scenarios as complex (preterm baby with pneumoperitoneum, baby with cancerous lung mass, baby with benign lung mass, child with kidney cancer).

**Fig. 2:**
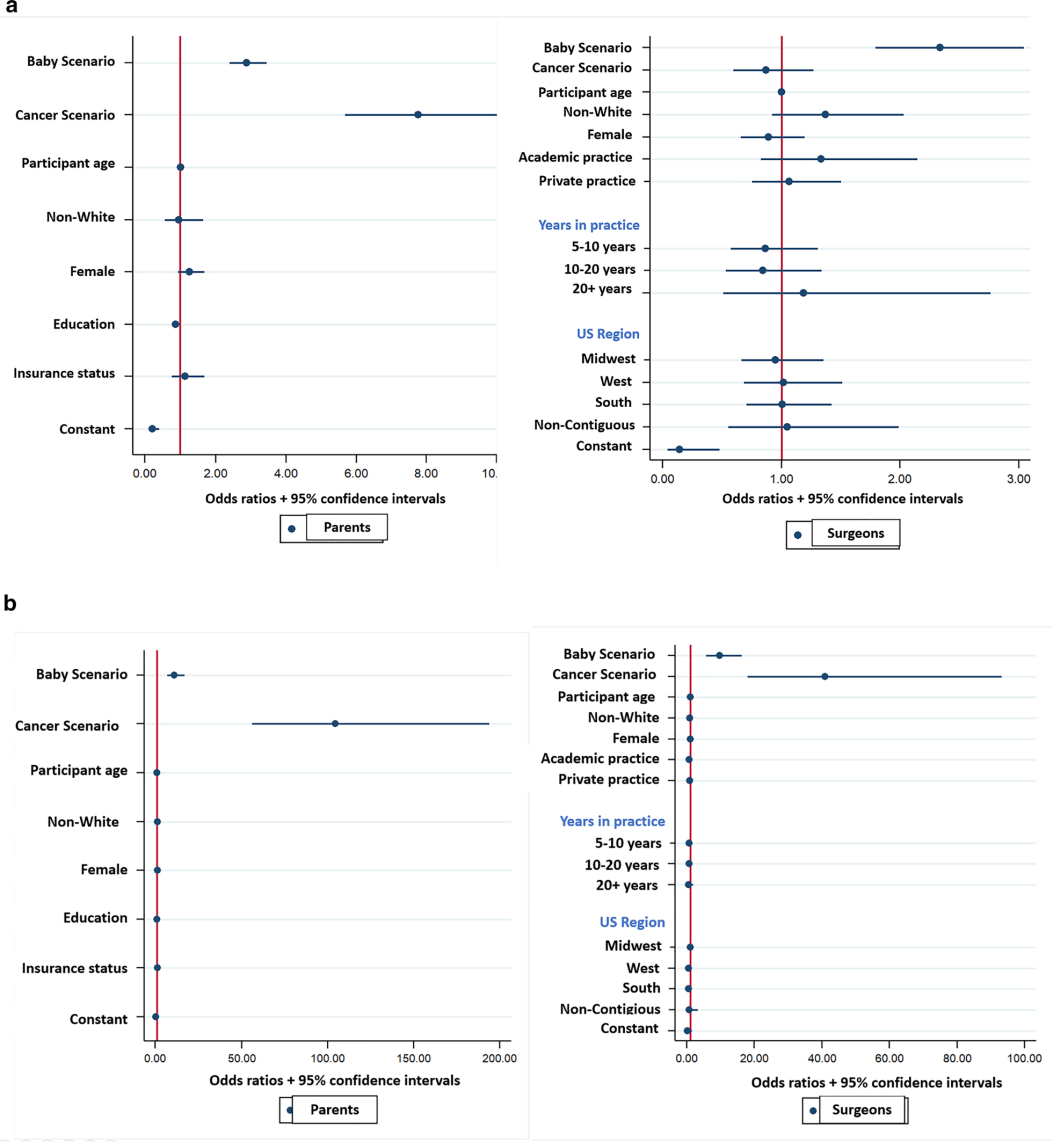
Odds ratios and 95% CIs for the association of rating a scenario as emergent (**a**) or complex (**b**) with scenario factors (age of patient, diagnosis) and demographic factors for parents and surgeons

Regarding level of urgency, scenarios were more likely to be rated emergent by both parents (P) and surgeons (S) when the patient was a baby compared to a child (P: 45.2% baby vs. 36.2% child, *p* = 0.001; S: 28.0% baby vs. 14.0% child, *p* < 0.001). However, when the diagnosis involved cancer, only parents were more likely to rate the scenario as emergent (P: 68.8% cancer vs. 29.5% non-cancer, *p* < 0.001; S: 19.2% cancer vs. 25.4% non-cancer, *p* = 0.051). Factors associated with increased odds of parents classifying a scenario as emergent included scenarios with a diagnosis of cancer (OR = 7.78, 95% CI 5.68–10.65, *p* < 0.001) or a baby (OR = 2.87, 95% CI 2.39–3.45, *p* < 0.001). For surgeons, only scenarios involving a baby were associated with increased odds of an emergent classification (OR = 2.34, 95% CI 1.79–3.05, *p* < 0.001) (Fig. 2a).

Regarding complexity, scenarios were more likely to be rated as complex by both parents and surgeons when the patient was a baby as compared to a child (P: 66.5% baby vs. 50.8% child, *p* < 0.001; S: 61.6% baby vs. 43.4% child, *p* < 0.001) or the diagnosis involved cancer (P 95.9% cancer vs. 44.1% non-cancer, *p* < 0.001; S: 86.7% cancer vs. 39.2% non-cancer, *p* < 0.001). Factors associated with increased likelihood of classifying a scenario as complex for both parents and surgeons included scenarios involving a cancer diagnosis (P: OR = 104.38, 95% CI 56.21–193.82, *p* < 0.001; S: OR = 40.94, 95% CI 17.97–93.24, *p* < 0.001) or a baby (P: OR = 10.90, 95% CI 7.00–17.06, *p* < 0.001; S: OR = 9.71, 95% CI: 5.76–16.35, *p* < 0.001) (Fig. 2b).

### Ranking of factors used by parents and surgeons to determine whether a scenario was emergent

**Fig. 3:**
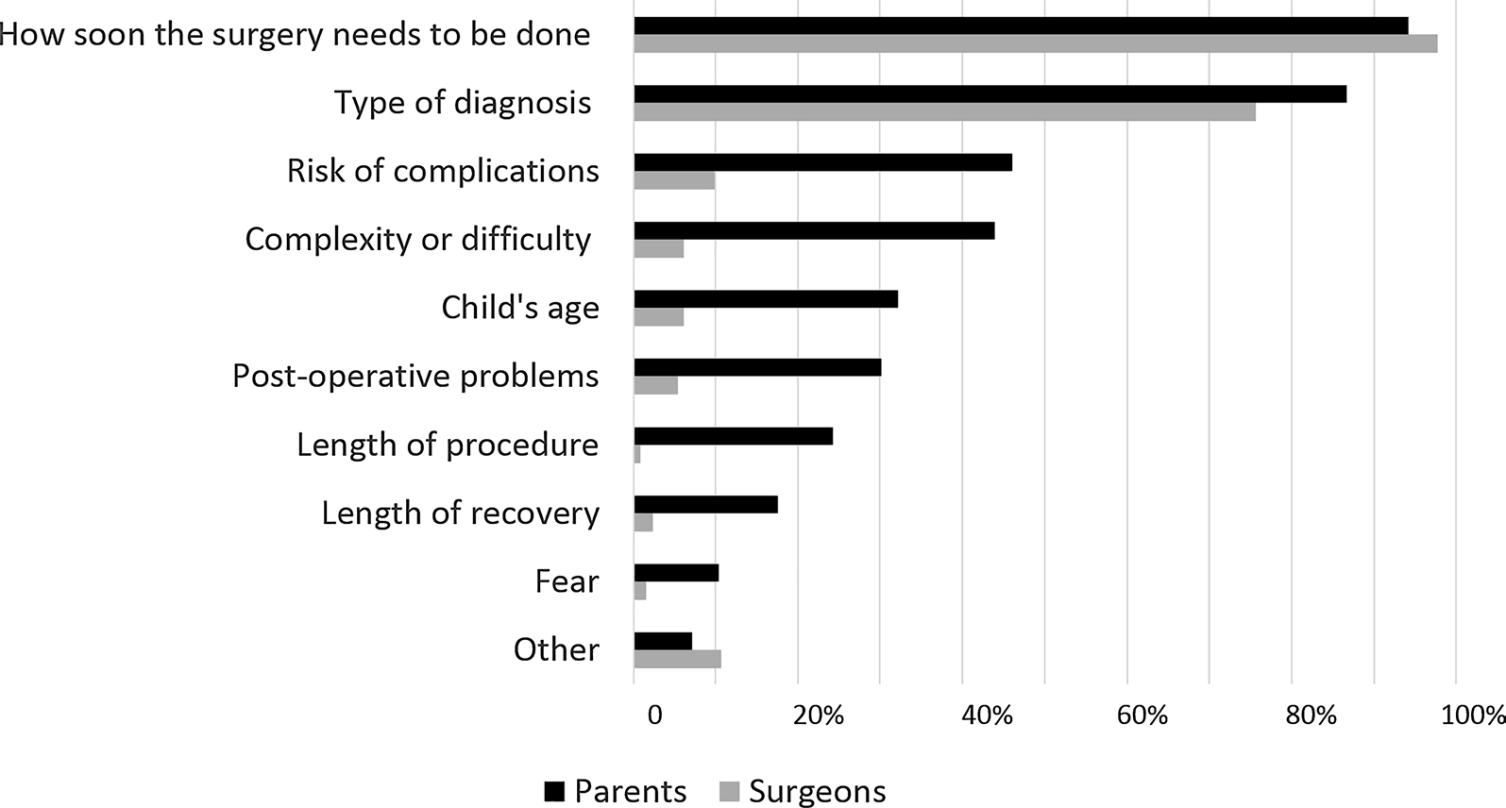
Factors considered important in determining if a given surgery is an emergency for parents as compared to surgeons

Most parents and surgeons identified “How soon the surgery needs to be done” as the primary factor in assessing whether a surgery is emergent (94% and 97% respectively). “Type of diagnosis” was the second most cited factor for both parents and surgeons (86% and 76% respectively). Though the ranking of factors used by surgeons and parents was similar, parents were more likely to include more factors in their assessment of urgency than were surgeons (Fig. 3).

### Impact of scenario factors, complexity, and perceived level of urgency on parents’ and surgeons’ decision-making preferences

**Fig. 4:**
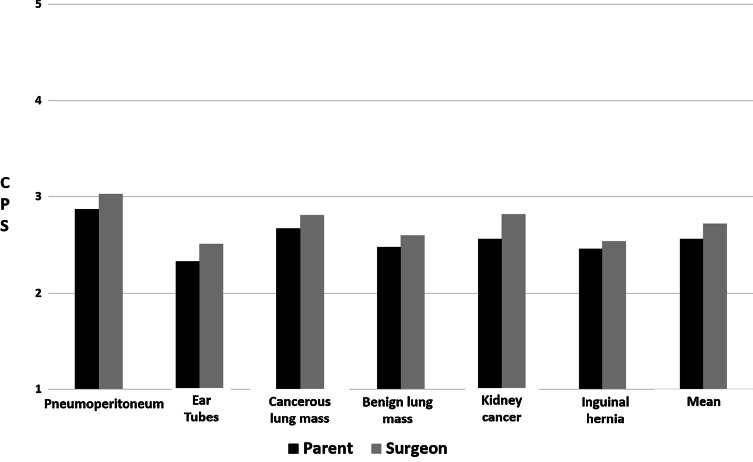
Average CPS score per scenario

**Fig. 5:**
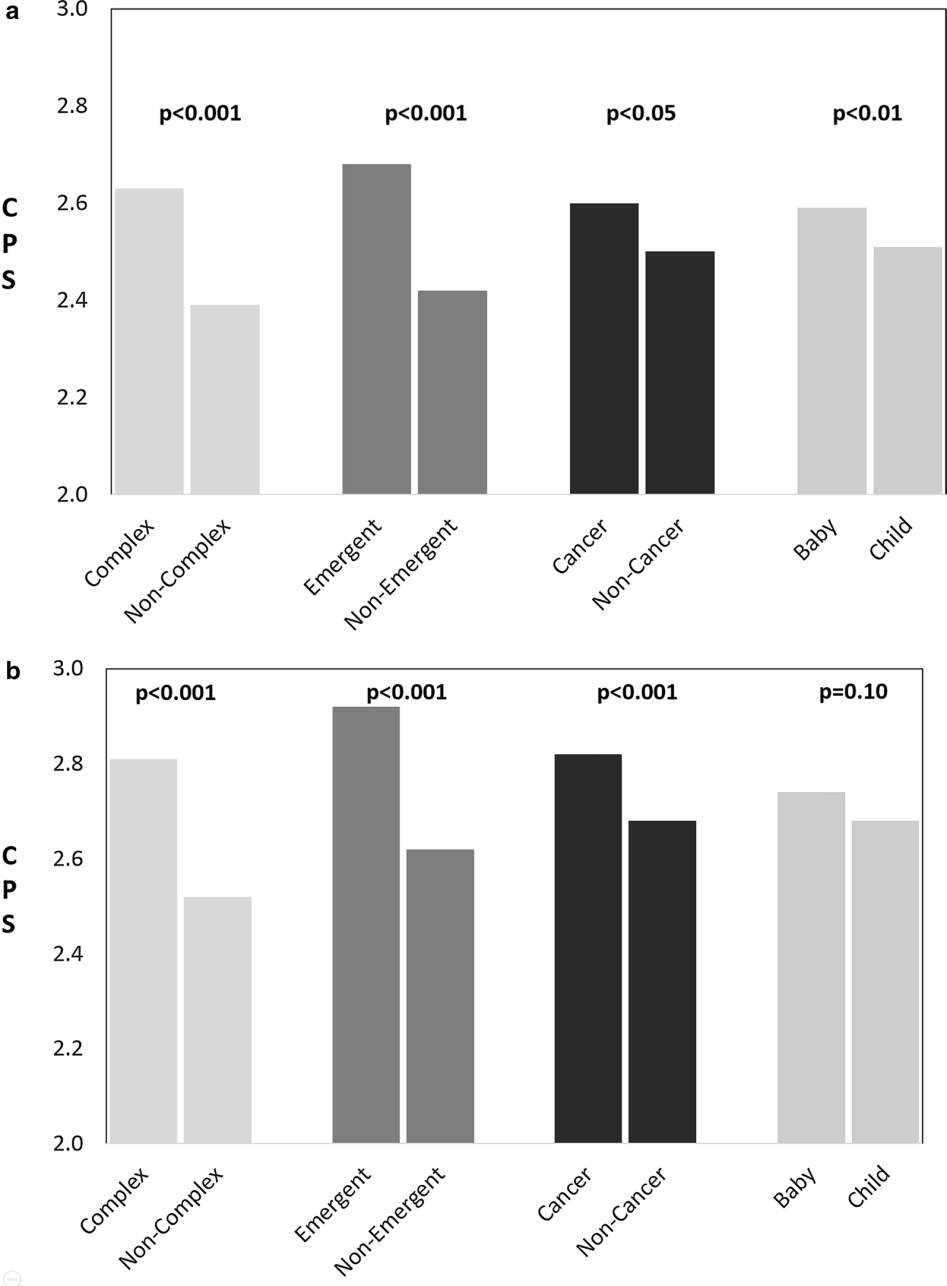
CPS scores for parents (**a**) and surgeons (**b**) for complex as compared to non-complex operations, emergent as compared to non-emergent operations, cancer as compared to non-cancer operations, and operations performed on babies as compared to children. Higher CPS scores suggest a preference for more surgeon guidance, while lower CPS scores suggest a preference for parent autonomy in decision-making

With reference to the CPS scale (1 = autonomous patient decision-making, 2 = patient-led with surgeon input, 3 = shared decision-making, 4 = surgeon-led with patient input, 5 = decision made by surgeon), parents had an average CPS score of 2.56 (95% CI 2.47–2.65; SD = 0.71) across the 6 scenarios (Fig. 4). Factors associated with a parental preference for more surgeon guidance included: complex cases (*p* < 0.001), emergent cases (*p* < 0.001), cancer diagnoses (*p* = 0.02), and scenarios involving babies (*p* < 0.01) (Fig. 5a). Respondents with at least a college degree had lower average CPS score than those without a college degree (*p* < 0.05) (Fig. 5a).

Surgeons had an average CPS score of 2.72 (95% CI 2.61–2.82; SD = 0.61) across the 6 scenarios (Fig. 4). Factors associated with surgeon preference for more surgeon guidance included: complex cases (*p* < 0.001), emergent cases (*p* < 0.001), and cancer diagnoses (*p* < 0.001). Scenarios involving babies did not have significantly different CPS scores than those involving children (*p* = 0.10). Surgeon demographic factors did not impact CPS scores (Fig. 5b).

**Fig. 6:**
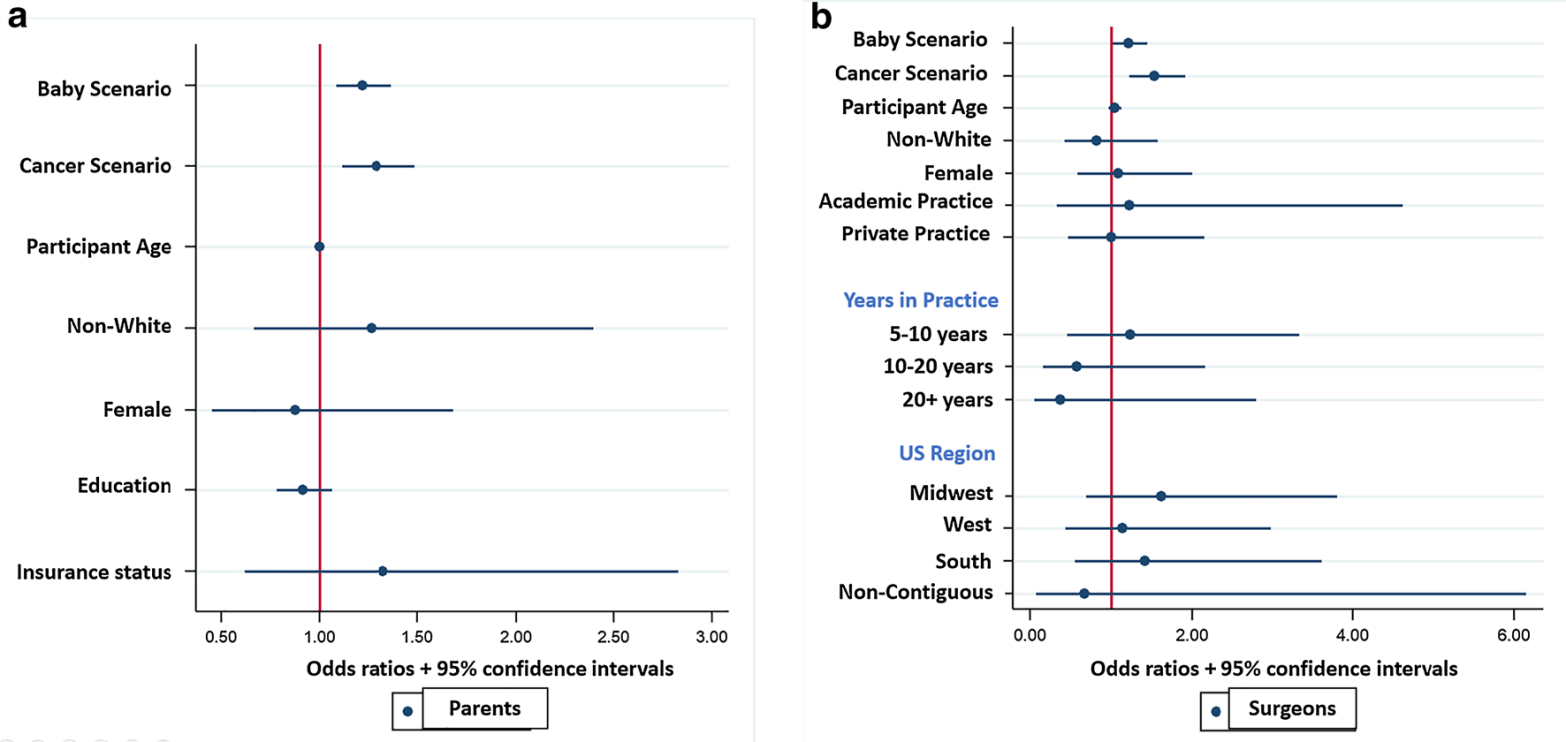
Odds ratios and 95% CIs for the impact of scenario factors (age of patient and diagnosis) and demographic factors on decision making preferences for parents (**a**) and surgeons (**b**)

Multivariable analysis including scenario factors as predictors demonstrated that for parents, baby scenarios and cancer scenarios were significantly associated with increased odds of decision-making preferences for more surgeon guidance (Baby: OR 1.22; 95% CI 1.08–1.37; *p* < 0.01; Cancer: OR 1.29; 95% CI 1.11–1.49; *p* < 0.01) (Fig. 6a). For surgeons, only cancer scenarios were significantly associated with increased odds of decision-making preferences for more surgeon guidance (OR 1.53; 95% CI 1.23–1.92; *p* < 0.001) (Fig. 6b).

**Fig. 7:**
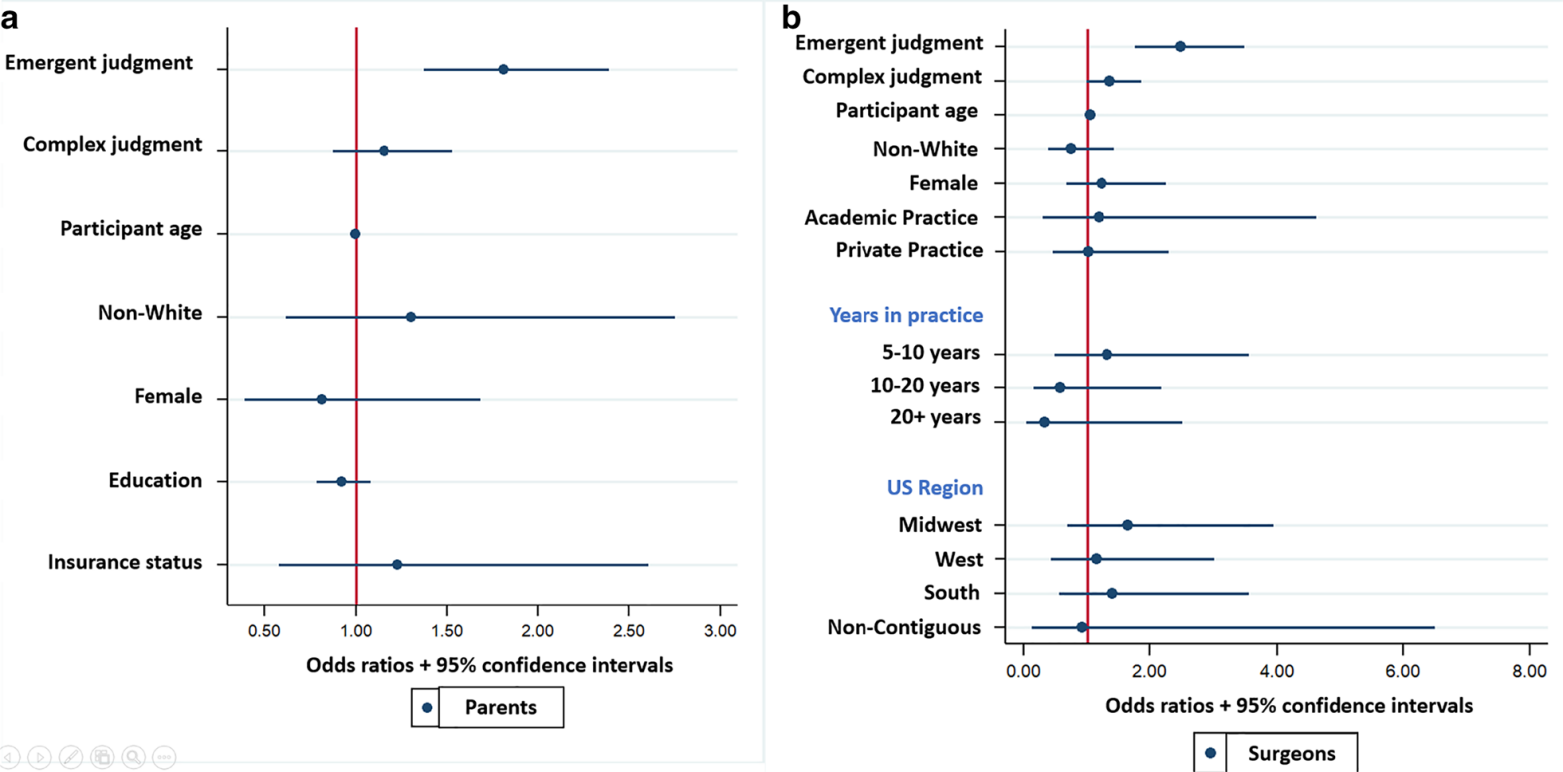
Odds ratios and 95% CIs for the impact of perceived urgency/complexity and demographic factors on decision making preferences for parents (**a**) and surgeons (**b**)

Multivariable analysis using participant assessments of the urgency and complexity of each scenario demonstrated that for both parents and surgeons, an emergent rating (but not a complex rating) was associated with increased odds of a preference for more surgeon guidance (P: OR 1.81; 95% CI 1.37–2.38, *p* < 0.001; S: OR 2.48 95% CI 1.76–3.49, *p* < 0.001) (Fig. 7a, b).

## Discussion

While many assert that SDM is the preferred approach to clinical counseling [1, 2], others express concern that patients and families may prefer more guidance from physicians than the traditional concept of SDM recommends [4, 5, 7]. Our work suggests that overall, both parents and surgeons endorse some form of SDM. However, SDM does not seem to be a uniform construct, but rather a concept that exists along a continuum ranging from less surgeon guidance to more surgeon guidance. In general, parents seemed to prefer more guidance when they believed a situation to be urgent. Interestingly, parent’s perceptions of urgency sometimes differed from those of surgeons. Awareness of parent’s perceptions can help surgeons adjust their level of directiveness to meet the needs of parents in a given clinical encounter.

Notably, we found that when a pediatric patient was diagnosed with cancer, parents were more likely than surgeons to perceive the situation to be emergent, thereby suggesting that they would prefer more guidance from their surgeon during counseling regarding surgery for pediatric cancer. Additionally, we found that while surgeons did not express the need to offer parents a different level of guidance based upon patient age (baby vs. child), parents did make an age-based distinction, preferring more guidance from surgeons when the patient was a baby as compared to a child. These findings suggest that parental perceptions of the level of urgency of a given clinical setting impact their desired level of guidance from surgeons. This suggests that situations may arise in which there is a mismatch between the level of guidance parents desire and the level of guidance their surgeons think they want. Such discrepancies may impact parental satisfaction with surgical counseling as well as parental confidence in the surgeon. If surgeons recognize the possibility of such discrepancies, they will be in a better position to assess parents’ needs and consider shifting toward a more guided approach when discussing operative interventions for babies or children with cancer.

Relatively few studies specifically investigate situations in which a mismatch between parent and surgeon decision making preferences exists. A recent exploration of parent and clinician attitudes toward parental involvement in decision making regarding surgical placement of an intracranial pressure monitor in children who have undergone acute traumatic brain injury demonstrates that in this setting, both parents and providers prefer providers make the decision regarding whether to proceed with the procedure [7]. While this work suggests that parents and surgeons may demonstrate concordant preferences in some settings, further investigation is needed to better understand the factors that guide these preferences as well as clinical settings in which discordant preferences exist.

In light of the discrepancies in decision-making preferences between parents and surgeons identified in our work it is important to consider how parents and surgeons determine if a given situation is urgent. We found that both parents and surgeons relied on two key factors to decide if a situation is emergent: “How soon the surgery needs to be done” and “the type of diagnosis.” However, parents were more likely than surgeons to include other factors as well, such as risk of morbidity and mortality, complexity of the surgery, age of the child, and potential post-operative complications. Enhanced awareness of the need to consider these additional factors in parents’ assessment of urgency may help surgeons frame the informed consent process in a way that more accurately addresses parental concerns.

Overall, parents and surgeons reported CPS scores of 2 or 3 for most scenarios. A score of 2 implies that the parent prefers to use the surgeons’ recommendation to guide decision-making and that the surgeon prefers to offer the parent a recommendation to guide the parents’ decision. A score of 3 implies that shared decision-making is preferred. Neither parents nor surgeons ever selected purely autonomous decision-making (CPS score 1), suggesting that at least some level of guidance during decision-making is always preferred by both parties. This finding prompts one to question those who assert that any form of recommendation from a physician represents an intrusion on a patient’s autonomy [19], and it supports the suggestion that parents may want and need more physician guidance during decision-making [6, 8]. Our results also suggest that the parental desire for increased surgeon guidance may be influenced by parental education level. Though not confirmed by multivariable analysis, on univariable analysis parents with a college education or higher preferred more autonomous decision-making than those with less than a college education.

While the finding that both parents’ and surgeons’ CPS scores clustered around shared decision-making (CPS score 3) may simply suggest that they both prefer some degree of SDM, one must also consider that these results may have been influenced by social desirability bias. Surgeons have increasingly been taught that shared decision-making is the preferred approach to clinical counseling, and patients have been educated to be active participants in healthcare decision-making. These influences may have encouraged greater inclinations toward shared decision-making. But we also noted some differences in our data set between CPS scores of 2 and 3. Further work is needed to explore the nuances between what it means to engage in shared decision-making (CPS score 3) and what it means to use the surgeons’ recommendation to guide autonomous decision-making (CPS score 2). Qualitative methods would help elucidate this difference and may help refine the Controlled Preferences Scale thereby facilitating a more accurate understanding of decision-making preferences in future studies. Such accuracy is important, since many studies addressing decision-making in healthcare rely on this scale [16].

Development of qualitative investigations in pediatric oncology may be especially important given our finding that parents and surgeons have discrepant interpretations of the urgency of oncologic diagnoses. Increased understanding of these critical issues will help elucidate the most efficacious approach to counseling in pediatric surgery more generally. Assuring that appropriate guidance is offered should strengthen the relationship between parents and surgeons and promote the development of trust, which has been shown to predict adherence to and satisfaction with treatment plans [20]. Enhanced understanding of effective surgical counseling will also provide a framework to improve education that prepares surgical and proceduralist trainees for clinical counseling in difficult settings.

Our study has several limitations. The web-based nature of our survey likely resulted in a selection bias of respondents resulting in predominantly well-educated, privately-insured, white women in the parent group. Based on our low response rates for both parents and surgeons, our data may not be representative of patients/parents at the University of Iowa or pediatric surgeons in the United States; as a result, it may be appropriate to consider our respondent populations as convenience samples. Additionally, parent perspectives were gathered from a single, Midwestern, academic center, so the results may not be generalizable across institutions and regions. Furthermore, only surgeons practicing in the United States were surveyed. Inclusion of pediatric surgeons worldwide would likely provide a less culturally-biased sample. Further, our results may have been impacted by potentially confounding clinical variables within our scenarios. Despite efforts to construct relatively simple scenarios that facilitated direct comparisons (baby with cancerous lung mass v baby with benign lung mass), the clinical variables we focused on (baby and cancer) may merely be surrogates for clinical settings in which parents generally feel uncomfortable. Thus, attributing our results to these specific variables may not be entirely straightforward. The hypothetical nature of the scenarios may also have impacted our results. Future work will involve assessment of parent’s reflections upon pediatric surgical situations that they have previously encountered. Our work was also limited by reliance on a single item (CPS score) to measure decision making preferences. Inclusion of multiple scales would offer more robust insight into decision making preferences.

## Conclusion

The results of our study provide information that can improve clinical counseling in pediatric surgery -and, by extension, in pediatrics more broadly. To our knowledge, this is the only study that has examined parents’ and surgeons’ perceptions of the level of urgency and complexity of a given clinical setting and the impact of these perceptions on the desired level of guidance from surgeons. We found that overall parents and surgeons value some level of shared decision making and demonstrate generally congruent interpretations of the urgency and complexity of various pediatric surgical issues. However, when a pediatric patient is diagnosed with cancer, parents are more likely than surgeons to deem this an emergent situation. This discordance is important to bear in mind, given that our findings also indicate that parents and surgeons prefer to shift toward more surgeon guidance when making decisions in emergent situations.

While further investigation is needed to refine our understanding of decision-making preferences, our results suggest that pediatric surgeons should be prepared to offer more guidance to parents when decisions need to be made in settings that are perceived to be emergent or involve a diagnosis of cancer. Our findings further suggest that surgeons must remain mindful of other potential clinical settings in which parent’s desired level of guidance may differ from what surgeons anticipate. Heightened awareness of this possibility will allow surgeons to be better prepared to pivot toward a more guided approach to clinical counseling in such settings. Assuring that the appropriate amount of guidance is provided may enhance parent satisfaction with the clinical encounter and trust in the surgeon thereby fostering the development of a collaborative partnership well poised to optimize the delivery of patient care.

## Supplementary Information

**Additional file 1.** Appendix A and B. Parent and surgeon surveys.

## Data Availability

The datasets used and/or analyzed during the current study are available from the corresponding author on reasonable request.

## Abbreviations

CPS: Controlled Preferences Score
SDM: Shared Decision Making
APSA: American Pediatric Surgical Association
