# Supplementary material for: Discrepancies in decision making preferences between parents and surgeons in pediatric surgery
Source: BMC Med Inform Decis Mak. 2021 Feb 4;21:42. doi: 10.1186/s12911-021-01414-z (PMC7863410; doi:10.1186/s12911-021-01414-z)
Supplement: Supplementary file 1 — Additional file 1. Appendix A and B. Parent and surgeon surveys. [file 12911_2021_1414_MOESM1_ESM.docx]

Discrepancies in decision making preferences between parents and surgeons in pediatric surgery

Erica M Carlisle MD^a,b,c^, Caleb J Klipowicz MA^d^, Laura A Shinkunas MS^b^, Aaron M Scherer PhD^a^, Lauris C Kaldjian MD PhD^a,b^

**Affiliations:**

^a^University of Iowa Carver College of Medicine

^b^University of Iowa Carver College of Medicine, Program in Bioethics and Humanities

^c^University of Iowa Hospitals and Clinics, Department of Surgery, Division of Pediatric Surgery

^d^ University of Iowa, Department of Anthropology

**Corresponding author at:**

Erica M. Carlisle MD

Assistant Professor of Surgery and Pediatrics

Affiliate Faculty in the Program in Bioethics and Humanities

University of Iowa

200 Hawkins Drive 2966-Z-JPP

Iowa City, IA 52242 USA

Phone: 319.356.1766

Fax: 319.384.9610

Email: [erica-carlisle@uiowa.edu](mailto:erica-carlisle@uiowa.edu)

**Appendix A. Parent survey**

**Scenarios:**

A. You are the parent of a premature baby born at 30 weeks gestation (typical birth is 40 weeks gestation). Your baby has been doing well in the neonatal intensive care unit. However, today the doctors noticed that your baby’s abdomen is bloated. Your baby also had a bloody bowel movement and looks uncomfortable. An abdominal X-ray shows signs that the intestine has perforated (developed a hole that is leaking). This can make your baby very sick and result in a risk of dying within hours or days. The pediatric surgeon has evaluated your baby and is discussing surgery to repair the hole in the intestine with you. The surgeon tells you that the surgery should be done within the next hour, will take about 3 hours, may be associated with a high risk of complications, and may require weeks to months in the hospital for recovery.

B. You are the parent of a healthy 9 month old baby. Your baby has had 4 ear infections in the past 6 months, and the pediatrician advises that your baby undergo surgical placement of ear tubes to help drain the infection. The surgeon tells you that the surgery will require about 30 minutes and your baby will be able to go home the same day. The surgeon recommends that the surgery be done sometime in the next 4 weeks and tells you that there is a low risk of complications from surgery.

C. You are the parent of a six month old baby who has been diagnosed with a 2 inch mass in the right lung. Biopsy of the mass shows that it is cancer. Your baby’s doctors have told you that chemotherapy should be completed (which will take 4 weeks) prior to surgery to remove the mass. Your baby’s survival depends on how well the chemotherapy shrinks the mass and whether the surgeon is able to remove all of the mass during the operation. You are meeting with the surgeon in clinic today to discuss the operation. The surgeon tells you that the surgery will take about 4 hours, your baby will stay in the hospital for about 5 days after surgery, and there is a risk of significant bleeding and injury to the large blood vessels in the chest as well as a risk of injury to the lungs. The surgeon recommends that the surgery be done soon (about a week) after chemotherapy is completed.

D. You are the parent of a 3 month old baby with a 2 inch cystic mass in the right lung. The mass was initially seen on a prenatal ultrasound, and physicians believe it is not cancer. Your baby had an uncomplicated birth, has not had any problems with breathing, was discharged home from the hospital the day after birth, and has been doing very well since. You are seeing the surgeon in clinic today to discuss surgery to remove the mass. The surgeon tells you that the mass should be removed because it has a risk of becoming infected when your baby has colds. The surgeon recommends that the surgery be done within the next 2-3 months. The surgeon tells you that the surgery will take about 3 hours, your baby will be in the hospital for 2-3 days after surgery, and there is a low risk of complications.

E. You are the parent of a 3 year old child who has always been healthy. During a bath, you notice that your child’s abdomen is more bloated than usual. You bring your child to the pediatrician who performs an ultrasound. The ultrasound shows that there is a mass in your child’s kidney that is most likely cancer. You are meeting with the pediatric surgeon in clinic today to discuss surgery to remove the mass. The surgeon tells you that the surgery should be done within the next 2 weeks. The surgeon expects that this surgery will take 4 hours to complete and tells you that your child will need to stay in the hospital for about 5 days after the operation. Your child may need chemotherapy or radiation treatment after surgery.

F. You are the parent of a healthy 5 year old child. Over the past week you have noticed that your child has a lump in the groin. The lump does not seem to hurt your child, and it does not seem to be causing any other problems. You bring your child to the pediatrician for a check-up. The pediatrician thinks that the lump is a hernia and refers you to a pediatric surgeon to discuss surgery to repair the hernia. The surgeon tells you that the surgery will take about one hour, and your child will be able to go home the same day. The surgeon recommends that the surgery be performed within the next 4 weeks and tells you that there is a low risk of complications.

**Questions for each scenario:**

Do you think this surgery is an emergency? Yes No I don’t know

Do you think this surgery is complex or difficult to perform? Yes No I don’t know

Although the doctors have recommended surgery for your child, parents must give consent for surgeries before they are performed. How would you prefer to make the decision about surgery for your child?

A. I would prefer to make the decision about surgery after getting the necessary information from the surgeon.

B. I would prefer to make the decision about surgery after getting the necessary information from the surgeon and considering the surgeon’s recommendation.

C. I would prefer to work together with the surgeon to make the decision about surgery.

D. I would prefer to let the surgeon make the decision about surgery after telling the surgeon my opinion about the surgery.

E. I would prefer to let the surgeon make the decision about surgery, and I would not need to tell the surgeon my opinion about the surgery.

**Question at the End of the survey:**

What issues do you consider when determining if a given surgery is an emergency? (check all that apply)

Type of diagnosis (cancer, infection, etc)

Length of time surgery will take

Amount of problems the child may have after surgery

How soon the surgery needs to be done (hours, days, weeks, months)

How high the risk of complications is for the surgery

The age of the child

The complexity or difficulty of the surgery

My own fear about the surgery

The length of recovery following surgery

Other: (free text)

**Appendix B. Surgeon survey**

**Scenarios:**

A. You are consulted on a premature baby born at 30 weeks gestation. The baby had been doing well in the neonatal intensive care unit. However, today the doctors noticed that the baby’s abdomen is bloated. The baby also had a bloody bowel movement and looks uncomfortable. An abdominal X-ray shows signs that the intestine has perforated. This can make the baby very sick and result in a risk of dying within hours or days. You have evaluated the baby and are discussing surgery to repair the hole in the intestine with the baby’s parent/guardian. You tell the parent/guardian that the surgery should be done within the next hour, will take about 3 hours, may be associated with a high risk of complications, and may require weeks to months in the hospital for recovery.

B. You are consulted on a healthy 9 month old baby. The baby has had 4 ear infections in the past 6 months, and the pediatrician advises that the baby undergo surgical placement of ear tubes to help drain the infection. You tell the parent/guardian that the surgery will require about 30 minutes and the baby will be able to go home the same day. You recommend that the surgery be done sometime in the next 4 weeks and tell the parent/guardian that there is a low risk of complications from surgery.

C. You are consulted on a six month old baby who has been diagnosed with a 2 inch mass in the right lung. Biopsy of the mass shows that it is cancer. You have told the parent/guardian that chemotherapy should be completed (which will take 4 weeks) prior to surgery to remove the mass. The baby’s survival depends on how well the chemotherapy shrinks the mass and whether you are able to remove all of the mass during the operation. You are meeting with the parent/guardian in clinic today to discuss the operation. You tell the parent/guardian that the surgery will take about 4 hours, the baby will stay in the hospital for about 5 days after surgery, and there is a risk of significant bleeding and injury to the large blood vessels in the chest as well as a risk of injury to the lungs. You recommend that the surgery be done soon (about a week) after chemotherapy is completed.

D. You are consulted on a 3 month old baby with a 2 inch cystic mass in the right lung. The mass was initially seen on a prenatal ultrasound, and you believe it is not cancer. The baby had an uncomplicated birth, has not had any problems with breathing, was discharged home from the hospital the day after birth, and has been doing very well since. You are seeing the parent/guardian in clinic today to discuss surgery to remove the mass. You tell the parent/guardian that the mass should be removed because it has a risk of becoming infected when the baby has colds. You recommend that the surgery be done within the next 2-3 months. You tell the parent/guardian that the surgery will take about 3 hours, the baby will be in the hospital for 2-3 days after surgery, and there is a low risk of complications.

E. You are consulted on a 3 year old child who has always been healthy. During a bath, the child’s parent/guardian notices that the child’s abdomen is more bloated than usual. The parent/guardian brings the child to the pediatrician who performs an ultrasound. The ultrasound shows that there is a mass in the child’s kidney that is most likely cancer. You are meeting with the parent/guardian in clinic today to discuss surgery to remove the mass. You tell the parent/guardian that the surgery should be done within the next 2 weeks. You expect that this surgery will take 4 hours to complete and tell the parent/guardian that the child will need to stay in the hospital for about 5 days after the operation. The child may need chemotherapy or radiation treatment after surgery.

F. You are consulted on a healthy 5 year old child. Over the past week the parent/guardian has noticed that the child has a lump in the groin. The lump does not seem to hurt the child, and it does not seem to be causing any other problems. The parent/guardian brings the child to the pediatrician for a check-up. The pediatrician thinks that the lump is a hernia and refers the child to you to discuss surgery to repair the hernia. You tell the parent/guardian that the surgery will take about one hour, and the child will be able to go home the same day. You recommend that the surgery be performed within the next 4 weeks and tell the parent/guardian that there is a low risk of complications.

**Questions for each scenario:**

Do you think this surgery is an emergency? Yes No I don’t know

Do you think this surgery is complex or difficult to perform? Yes No I don’t know

How would you prefer the decision about surgery be made for this child?

A. I would prefer the parent/guardian make the decision about surgery after getting the necessary information from me.

B. I would prefer the parent/guardian make the decision about surgery after getting the necessary information from me and considering my recommendation.

C. I would prefer to work together with the parent/guardian to make the decision about surgery.

D. I would prefer to make the decision about surgery after getting the parent/guardian’s opinion about the surgery.

E. I would prefer to make the decision about surgery without getting the opinion of the parent/guardian.

**Question at the End of the survey:**

What issues do you consider when determining if a given surgery is an emergency? (check all that apply)

Type of diagnosis (cancer, infection, etc)

Length of time surgery will take

Amount of problems the child may have after surgery

How soon the surgery needs to be done (hours, days, weeks, months)

How high the risk of complications is for the surgery

The age of the child

The complexity or difficulty of the surgery

My own fear about the surgery

The length of recovery following surgery

Other: (free text)
